# Serum amino acid profile in 51 dogs with immunosuppressant-responsive enteropathy (IRE): a pilot study on clinical aspects and outcomes

**DOI:** 10.1186/s12917-020-02334-2

**Published:** 2020-04-22

**Authors:** Elena Benvenuti, Alessio Pierini, Eleonora Gori, Francesco Bartoli, Paola Erba, Pietro Ruggiero, Veronica Marchetti

**Affiliations:** 1 Department of Veterinary Science, University of Pisa, via Livornese, 56122 San Piero a Grado, Pisa, Italy; 2 Department of Translational Research and New Technologies in Medicine and Surgery, University of Pisa, Via Savi, 10, Pisa, 56126 Italy; 3 Professional Association Endovet Rome, Rome, Italy

**Keywords:** Tryptophan, Histidine, CCECAI, Canine, Body condition score

## Abstract

**Background:**

Lower levels of tryptophan (TRP) have been identified in people with inflammatory bowel disease and in dogs with protein-losing enteropathy (PLE). No data on serum amino acids (AAs) but some on plasma in canine immunosuppressant-responsive enteropathy (IRE) are available. The aim of this study is to compare serum AAs between healthy and IRE dogs, considering clinicopathological variables and follow-up.

**Results:**

Twenty-six healthy control dogs (CD) and 51 IRE dogs were included. IRE was diagnosed after the exclusion of extra-intestinal diseases and food and antibiotic responsive enteropathies. The canine chronic enteropathy clinical activity index (CCECAI) was assessed at presentation and during the clinical follow-up. In CD and IRE dogs, 19 different serum AAs were measured. IRE dogs were classified into responders, partial responders and non-responders, based on CCECAI after 1 month, and divided into PLE and non-PLE, based on albumin level. IRE dogs showed lower L-Tyrosine (TYR), L-Phenylalanine (PHE) and TRP (*p* < 0.001) and higher L-Serine (SER), L-Glutamic acid (GLU), L-Arginine (*p* < 0.001), L-Threonine (*p* = 0.013), Proline (*p* = 0.044), L-Cysteine (*p* = 0.003), L-Valine (*p* = 0.018), L-Lysine (*p *= 0.01) and L-Isoleucine (*p* = 0.005) than CDs. PLE dogs showed lower L-Histidine (HIS) (*p* = 0.008), PHE (*p* = 0.005) and TRP (*p* = 0.005) than non-PLE dogs. In IRE dogs, median GLU was significantly lower in dogs with BCS 3/9 than BCS 5/9 category (*p* = 0.036). Total protein was positively correlated with PHE and TRP (both *p* = 0.031, r = 0.30) and albumin was positively correlated with HIS (*p* = 0.025, r = 0.31), PHE and TRP (both *p* = 0.001, r = 0.46). HIS (*p* = 0.041), PHE (*p* = 0.047) and TRP (*p* = 0.044) concentrations were significantly lower in non-responders than in responders and partial responders.

**Conclusions:**

This study may suggest further investigation on serum, HIS, PHE, TRP and TYR as markers of intestinal disease and proposed HIS, PHE and TRP as prognostic marker for response to therapy.

## Background

Amino acids (AAs) are required for gastrointestinal health, as they can improve intestinal growth and maintain mucosal integrity and barrier function [1]. In human inflammatory bowel disease (IBD), specific alterations in the metabolism of AAs can result from the pathophysiological process caused by bowel inflammation and can influence the progression of IBD [2, 3]. AAs have various functions, and can reduce oxidative stress, contribute to the restoration of mucosal homeostasis, balance inflammatory and proinflammatory cytokines production and increase immune regulatory cytokine concentration [1, 4, 5]. The microbiota synthesizes the different proteins and a variety of metabolites originating from AAs, which thus play an important role in the nutrition and physiology of the host [6].

In human gastroenterology, the evaluation of serum AAs is a non-invasive, predictive marker of intestinal inflammation, and the supplementation of AAs can attenuate this inflammation and improve clinical signs and weight gain [7, 8].

The immunosuppressant-responsive enteropathy (IRE) describes the intestinal idiopathic inflammation in dogs, that typically implies failed treatment trials with diet and subsequently antibiotics. In IRE, the intestinal inflammation has to be demonstrated by histological examination and requires an immunosuppressant treatment [9–11].

In veterinary medicine, few studies have examined AAs in dogs with chronic enteropathies [5, 12–14]. In dogs with protein-losing enteropathy (PLE), serum tryptophan (TRP) concentration has been found to significantly decrease compared with healthy dogs. TRP has also been positively correlated with serum albumin [5]. In a recent study, 21 plasma AAs were compared between 12 healthy dogs and 10 dogs with IRE [12]. The IBD dogs showed a significant decrease of methionine, serine, TRP and proline compared to healthy dogs. In addition, the canine chronic enteropathy clinical activity index (CCECAI) at the time of diagnosis was negatively correlated with plasma serine [12].

We hypothesize that the profile of serum AAs can be different between dogs diagnosed with immunosuppressant-responsive enteropathy (IRE) and healthy control dogs (CD). We also hypothesize that the serum AA profile may be associated with the clinical score and the response to treatment in IRE dogs. Thus, the aims of the present study are to: (1) compare the serum AA levels between control and IRE dogs and (2) evaluate the relationship between these levels and age, sexual status, body condition score (BCS), total protein, albumin, CCECAI and response to treatment in IRE dogs.

## Results

Fifty-one dogs with a final diagnosis of IRE were enrolled. The mean age was 5.3 ± 3.7 years, 33 (64%) were males (1 neutered) and 18 were females (36%) (6 spayed). Ten dogs were mixed breeds and the rest were as follows: German Shepherd (10), Maltese (3), Dachshund (3), Pinscher (2), Rottweiler (2), Yorkshire Terrier (2), Jack Russel Terrier (2), Spanish Galgo (1), Cavalier King Charles Spaniel (1), Weimaraner (1), West Highland White Terrier (1), Cocker Spaniel (1), Bernese Mountain Dog (1), Bolognese (1), Springer Spaniel (1), Boxer (1), Dobermann (1), Pug (1), Small Italian Levriero (1), Swiss Pastor (1), Beagle (1), Irish Setter (1), Vizsla (1), and Basenji (1). Median BCS was 3.5 (range 2–6).

**Fig. 1:**
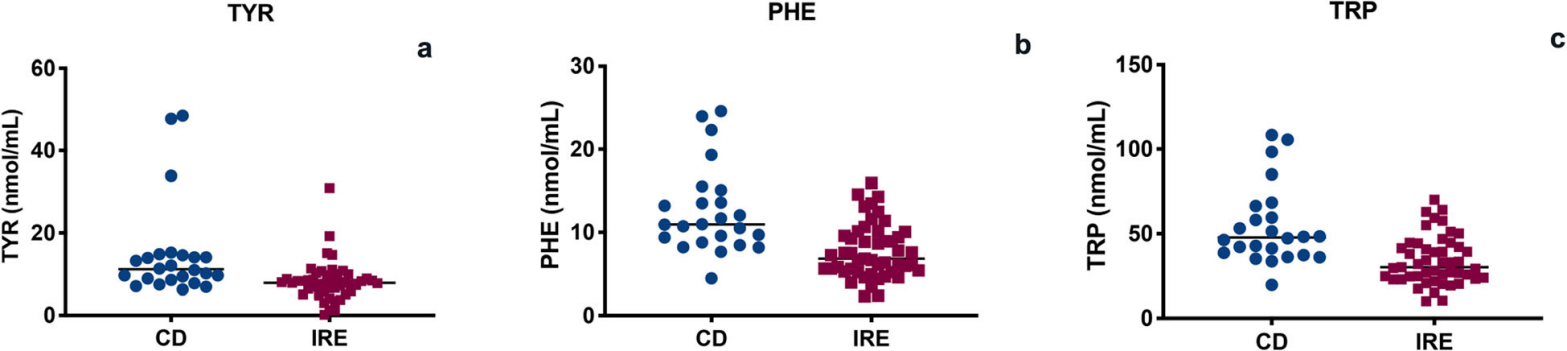
TYR (**a**), PHE (**b**) and TRP (**c**) serum evaluation in dogs with IRE and CD (*p* < 0.001)

The CD group was composed of 26 dogs with a mean age of 4.7 ± 3 years, and 12 were males (46%) (5 neutered) and 14 females (54%) (12 spayed). The CD included 8 mixed breeds and the following breeds: Dachshund (3), Labrador (2), Golden Retriever (2), English Setter (2), Jack Russel Terrier (2), Border Collie (1), Bernese Mountain Dog (1), Whippet (1), Maremma Shepherd (1), Cocker Spaniel (1), Poodle (1), and Lagotto Romagnolo (1). The median BCS was 4.5 (range 4–6). No difference in age and sex between the two study populations (IRE and CD) was found (*p* = 0.89 and *p* = 0.21, respectively). The median BCS of IRE dogs was significantly lower than in the CD dogs (median 4 range 2–6 vs. median 5 range 5–6; *p* < 0.0001). The IRE dogs showed significantly lower serum levels of TYR, PHE and TRP (*p* < 0.001) than the CD dogs (Fig. 1). However, the IRE group showed significantly higher levels of SER (*p* = 0.02), GLU (*p* < 0.001), ARG (*p* < 0.001), THR (*p* = 0.013), PRO (*p* = 0.044), CYS (*p* = 0.003), VAL (*p* = 0.018), LYS (*p* = 0.01) and ILE (*p* = 0.005) than the CD group. (see Table 1).

**Table 1:** Serum AA evaluation in dogs with IRE vs CD

Amino acid (nmol/mL)	CD (26 dogs)	IRE (51 dogs)	*p*-value
**AABA**^b^	34.63 ± 12.99	45.77 ± 15.89	0.06
**ALA**^a^	93.70 (56.57–225.9)	99.96 (14.23–334.1)	0.62
**ARG**^a^	57.80 (11.43–172.5)	84.54 (20.10–149.8)	**< 0.001**
**ASP**^a^	1.28 (0.16–5.46)	1.87 (0.21–12.08)	0.17
**CYS**^b^	39.90 ± 14.66	52.47 ± 18.22	**0.003**
**GLU**^a^	8.77 (4.38–20.55)	20.63 (4.32–67.85)	**0.001**
**GLY**^a^	48.44 (4.75–84.35)	48.92 (12.10–131.9)	0.64
**HIS**^a^	198.9 (24.9–323.4)	191.3 (21.3–326.9)	0.46
**ILE**^a^	23.84 (12.69–45.43)	29.94 (2.82–69.45)	**0.005**
**LEU**^a^	10.55 (7.76–19.40)	11.67 (1.77–20.18)	0.23
**LYS**^a^	7.16 (3.79–32.67)	9.56 (1.26–23.82)	**0.018**
**MET**^a^	4.77 (0.32–58.49)	5.96 (0.14–12.81)	0.21
**PHE**^b^	12.60 ± 5.24	7.73 ± 3.24	**< 0.001**
**PRO**^a^	6.15 (3.9–34.84)	11.02 (0.7–29.76)	**0.044**
**SER**^a^	35.50 (23–69.79)	45.01 (7.24–160)	**0.02**
**THR**^a^	48.78 (30.45–99.66)	68.29 (14.77–154.5)	**0.013**
**TRP**^a^	47.77 (19.68–108.3)	30.18 (10.24–70.18)	**< 0.001**
**TYR**^a^	11.15 (6.29–48.48)	7.87 (0.18–30.92)	**< 0.001**
**VAL**^a^	4.88 (2.96–12.68)	6.22 (0.64–62.49)	**0.018**

*CD* control group, *IRE* immunosuppressant responsive enteropathy, *AABA* L-Amino-n-butyric acid *ALA* Alanine, *ARG* L-Arginine, *ASP* L-Aspartic acid, *CYS* L-Cysteine, *GLU* L-Glutamic acid, GLY Glycine, *HIS* L-Histidine, *ILE* L-Isoleucine, *LEU* L-Leucine, *LYS* L-Lysine, *MET* L-Methionine, *PHE* L-Phenylalanine, *PRO* Proline, *SER* L-Serine, *THR* L-Threonine, *TRP* Tryptophan, *TYR* L-Tyrosine, *VAL* L-Valine. Non-parametric data are expressed as median and minimum and maximum in brackets and the respective p-values were obtained using Mann-Whitney U-test (^a^), while parametric data are expressed as mean ± standard deviation and respective *p*-values were obtained using unpaired t-test (^b^)

Among the IRE dogs, the serum AAs were not different between age categories and sex groups. Median GLU was significantly lower in dogs with BCS 3/9 (17.07 nmol/mL) than in the 5/9 BCS category (26.36 nmol/mL) (*p* = 0.036). No differences were observed between the other BCS categories.

**Fig. 2:**
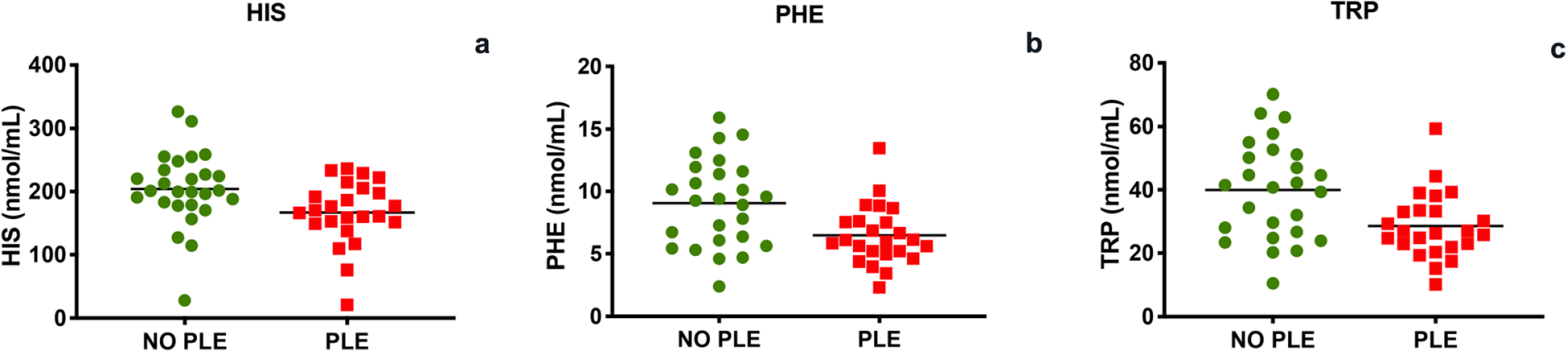
HIS (**a**), PHE (**b**) and TRP (**c**) serum evaluation in dogs with PLE and dogs with non-PLE (*p* < 0.05)

**Fig. 3:**
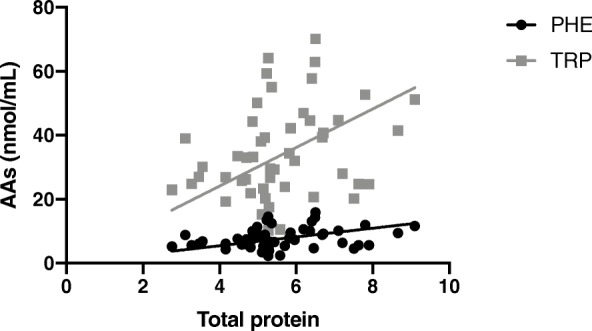
Correlation between TRP (*p* = 0.031, r = 0.3) and PHE (*p* = 0.031, r = 0.30) and total protein in IRE dogs

**Fig. 4:**
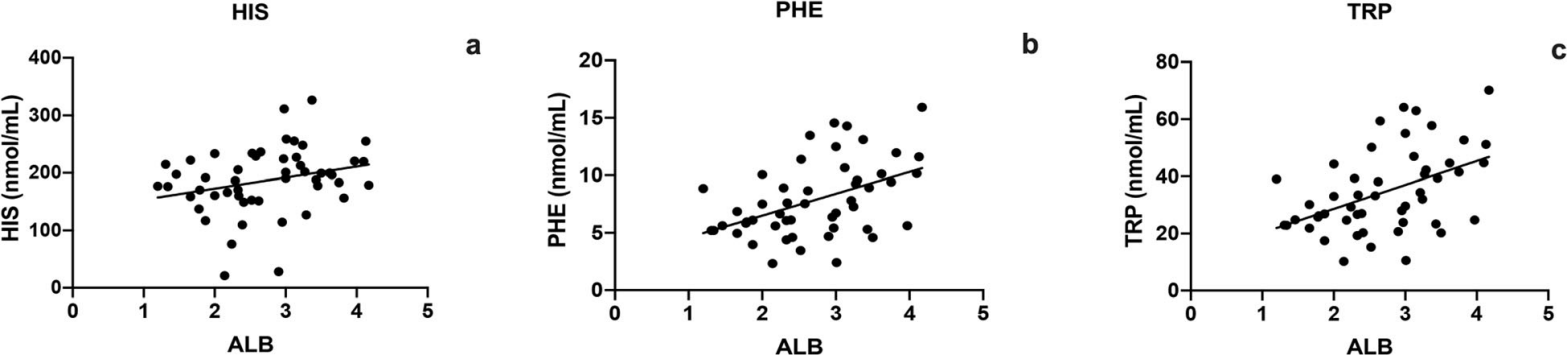
Correlation between HIS (**a**) (*p* = 0.025, r = 0.31), PHE (**b**) (*p* = 0.001, r = 0.46) and TRP (**c**) (*p* = 0.001, r = 0.46) and serum albumin in IRE dogs

The mean total protein of the serum was 5.6 ± 1.4 g/dL and the mean serum albumin 2.7 ± 0.8 g/dL; 26 (51%) dogs were diagnosed with PLE. The mean serum albumin concentration in PLE dogs was 2.1 ± 0.4 g/dL. In PLE dogs, PHE (*p* = 0.005), TRP (*p* = 0.005) and HIS (*p* = 0.008) were significantly lower than in non-PLE dogs (Fig. 2). In IRE dogs, serum total protein was positively correlated with PHE (*p* = 0.031, r = 0.30) and with TRP (*p* = 0.031, r = 0.30) (Fig. 3). In addition, serum albumin was positively correlated with PHE (*p* = 0.001, r = 0.46), TRP (*p* = 0.001, r = 0.46) and HIS (*p* = 0.025, r = 0.31) (Fig. 4).

**Table 2:** The elution gradient has the following parameters: HPLC gradient, the flow is kept constant at 1 ml/min

Time (min)	%A	%B	%C
0.0	100	0	0
0.5	99	1	0
18.0	95	5	0
19.0	91	9	0
29.5	83	17	0
33.0	0	60	40
36	100	0	0
50	100	0	0

*A* aqueous buffer, *B* acetonitrile HPLC grade, *C* water HPLC grade

At T0, the mean CCECAI score was 7 ± 3.6. None of the serum AAs was significantly correlated with CCECAI score.

**Fig. 5:**
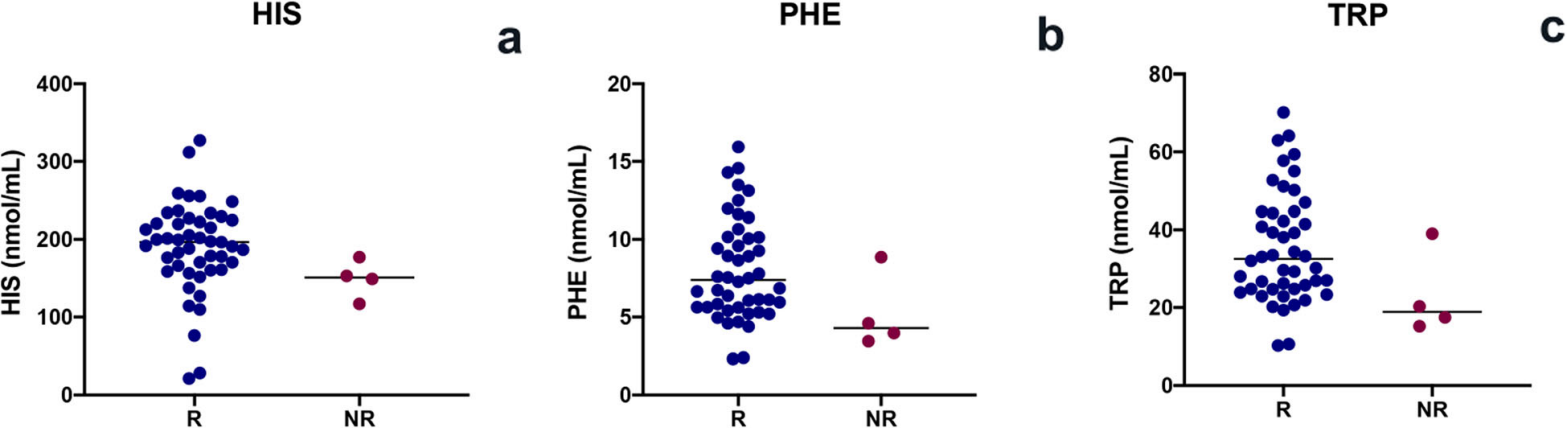
HIS (**a**), PHE (**b**) and TRP (**c**) serum concentration in responders (R) and non-responders (NR) (*p* < 0.05)

A total of 29 dogs were classified as complete responders, 18 as partial responders and 4 as non-responders. PHE (*p* = 0.047), TRP (*p* = 0.044) and HIS (*p* = 0.041) concentrations were significantly lower in non-responders than responders (Fig. 5). Nine dogs showed a relapse of disease during the six-month follow-up. Three of these dogs died due to the progression of their chronic enteropathy. The serum AAs showed no difference between relapsed and non-relapsed dogs.

## Discussion

In human medicine, the role of AAs in IBD and ulcerative colitis is well documented [4, 15, 16]. During human IBD, the integrity of the intestinal epithelial barrier can be impaired by the inflammatory process, thus causing pathogen penetration, malnutrition symptoms and general AAs deprivation which is associated with depression and muscle loss [4, 16–18]. Ulcerative colitis (UC) mice model showed decreases in several amino acids as TYR, glutamine, and alanine [19].

AAs are used as building blocks of macromolecule synthesis for intestinal mucosal and also play a major role in the intracellular protein turnover and energy substrates of enterocytes [3]. However, in veterinary medicine, AA modifications are rarely studied in dogs with chronic enteropathies [5, 12, 13]. In our study, dogs affected by IRE showed a lower serum TRP, PHE and TYR concentration than healthy dogs. A decrease of TRP has been reported in canine IBD and PLE [5, 12, 13] but we found that PHE and TYR are also decreased in the IRE dogs, as in humans.

This particular condition may reduce the absorption of AAs, with a reduction in TRP, PHE and TYR [4]. In addition, in human beings has been described that dysbiosis can alter AAs concentration during IBD, reducing their absorption [4, 19]. However, PHE and TYR deficiency has not as yet been described in canine IRE, although a pathogenic mechanism similar to that in human medicine can occur. Dogs with PLE likely also have a higher requirement for dietary protein because of increased demand from ongoing inflammation [5]. Furthermore, dogs with PLE can be unable to satisfy AAs requirements due to both intestinal AAs losses and to hypo/anorexia, which characterize this particular disease [5]. This latter hypothesis may explain why PHE and TRP levels were lower in our IRE dogs, with or without PLE. Many studies describe how integration of TRP, PHE, HIS and Glutamine in human IBD is an important part of therapy [1, 18, 20, 21], proposing TRP supplementation as an aid for the treatment of human IBD [1]. In fact, in humans, the TRP supplementation improves clinical signs, body weight, and intestinal permeability and reduces proinflammatory cytokines [1, 4, 20].

Currently, only one veterinary medicine study has described the use of L-Arginine and L-Glutamate for enhanced gastric motor function [21]. Although the effects of the integration of AAs in dogs with IRE are not yet established, supplementation may bring beneficial clinical effects in dogs with IRE, as in humans with IBD.

In our IRE dogs, ARG, LYS and PRO levels were higher than in healthy dogs. This has not been previously reported in veterinary medicine, but it has been described in humans. In humans, IBD has an impact on AA metabolism, showing increased levels of isoleucine (and its first degradation product 3-methyl-2-oxovalerate), MET, LYS, GLY, ARG, and PRO and decreased levels of VAL, TYR, and SER as compared to the control cohort [16, 19]. Especially MET, which is also a precursor of homocysteine, has been shown to be elevated in both plasma and colonic mucosa from IBD patients [19]. The tendency for AAs to increase in IBD patients may indicate increased de novo AAs synthesis maybe due to inflammation [22]. In fact, inflammatory conditions are known to stimulate protein catabolism and AAs release from muscle tissue and increased concentration of AAs might indicate a higher AAs turnover [14, 19].

Furthermore, we wanted to evaluate the possible relationship between AAs and clinical data, total proteins, albumin and clinical course of our IRE dogs, since this data is lacking in veterinary medicine literature.

Firstly, although all dogs in the IRE group were fed the same diet, GLU tended to be lower in dogs with BCS 3/9. GLU is a precursor for glutathione synthesis, and in IBD children, GLU showed significantly higher urinary levels than in the healthy controls. The higher urinary loss of GLU suggests that glutathione cannot be optimally synthesized and replenished, but the association with BCS remains unclear [23]. The modification of serum AAs in relation to BCS has not as yet been analyzed. There is a wide variability among breeds in body weight, body conformation, muscular mass and fat distribution [24]. These differences may potentially cause a variation in the metabolism of dietary AAs, as well as the concentration of each AA [24]. Although AAs concentration may be affected by diet and maybe time of fasting [5], we reduced both of these bias since all of the IRE dogs were fed the same hydrolyzed diet and were fasted for 8 h before the blood sampling.

In our study, serum HIS, PHE and TRP significantly decreased in dogs with PLE compared to non-PLE dogs. This finding supports those of Kathrani [5] but, as shown in our PLE cases, both PHE and HIS can be decreased. The TRP in dogs is an amino acid essential to diet, and is important for protein synthesis and is a precursor of kynurenine, serotonin, melatonin and picolinic acid. In Kathrani’s study, an increased expression of the intestinal enzyme indoleamine 2,3, dioxygenase 1 (IDO-1) and TRP catabolism in dogs with PLE was reported, which is similar to humans with IBD [5, 13, 25].

In human medicine the anti-inflammatory effect of HIS and PHE has been described. Both AAs cause a reduction of interleukin 6, 8 and TNFα levels, and their serum levels appear to be decreased in human with IBD and ulcerative colitis. The anti-inflammatory effects of HIS and PHE are dependent on its function in intestinal tight junctions [4, 26].

In dogs with PLE, the same pathological condition described in humans may occur, determining the reduction of HIS and PHE. In our study, we compared PLE vs. non-PLE dogs, unlike Kathrani et al. who considered PLE vs. healthy dogs. The differences in serum AAs concentration in PLE dogs, are therefore due to the different study population, since we compared IRE dogs with or without PLE. Our data show that in PLE dogs the serum decrease of HIS and PHE can be considered in addition to TRP reduction.

As PLE is considered a more severe form of intestinal inflammation [5], the positive correlation between HIS, PHE and TRP and albumin serum concentration can be considered as an indicator of the severity of loss from the gastrointestinal tract [5].

In human IBD, HIS and PHE appear to act as early markers of intestinal inflammation [26] and show a negative correlation with disease activity scores [2].

In our study, the AA serum levels were not associated with CCECAI score categories at T0. In veterinary studies, Tamura et al. described a significantly negative correlation between CCECAI score and plasma SER at the time of diagnosis [12]. Also, in another study, positive correlations were found between CCECAI scores and plasma VAL and between CCECAI scores and plasma ALA concentrations [14]. This was not unexpected, because AAs may differ depending on whether they are measured in serum or plasma [19]. This difference may be due to the release of mediators from platelets during the coagulation processes. The recent study by Yu et al. suggests that serum may be more sensitive to biomarker detection compared to plasma, whereas measuring AAs in plasma may be more reproducible [27].

So far, in veterinary medicine, no studies have investigated serum AAs concentrations and their relationship with follow-up in IRE dogs. In our study, non-responders showed lower serum TRP, PHE and HIS concentrations than responders. Since this is the first report which evaluated the AAs concentration related to response to treatment, a direct comparison with veterinary literature is not possible. However, we can only speculate about this data, since, at today, AAs are still a controversial topic about IRE dog. In our opinion, non-responder dogs may have a reduced in AAs absorption, especially of TRP, PHE and HIS, and maybe a concurrent dysbiosis, which can influence the serum concentrations of these particular AAs.

The limitations of this study include the difference in the number of IRE dogs and those in the CD, and the CD were fed a different diet, which may have influenced the results.

In human medicine, it has been shown that diet can influence microbiota composition and that the integration of AAs has positive effects [3]. Evaluating the microbiota composition in relation to AA serum concentration would also be of interest in veterinary medicine.

The serum AA evaluation was not repeated during follow-up, although it could give some practical information regarding the need or the benefit of their supplementation. Another limitation of the study is the lack of a muscular condition score evaluation. This parameter may lead to further interesting findings.

## Conclusion

In conclusion, this study may suggest further investigation on using serum TRP, PHE, HIS and TYR as markers of intestinal disease and some AAs (HIS, PHE and TRP) serum concentration might also be indicators of disease severity and response to therapy. Evaluating the effects of specific AAs integrations in further studies is essential.

## Methods

### *Clinical and case selection criteria*

This retrospective study was conducted at the Veterinary Teaching Hospital (University of Pisa) between February 2018 and January 2019. Each blood sample was collected from the dogs at presentation, together with other diagnostic investigations and a signed informed consent was obtained from their owners. Thus, a formal approval of the Institutional Committee for Animal Care was requested (number 31834/2017).

Dogs diagnosed with IRE were retrospectively included. The IRE diagnosis was conducted after the exclusion of extra-intestinal diseases, infectious or parasitic diseases and intestinal diseases of other etiology (e.g., mechanical obstruction from intussusception, foreign body or intestinal tumors). The dogs were included after the exclusion of food responsive enteropathy with hydrolyzed diet and antibiotic responsive enteropathy [9]. The histologic samples were collected by endoscopic biopsies in all dogs and the examination was performed according to the standard of the World Small Animal Veterinary Association Gastrointestinal Standardization Group [10, 11].

Each dog was fasted at least 8 h before blood sampling and serum total protein and serum albumin were also evaluated in all IRE dogs. Hypoproteinemia was defined as a total protein of < 5.8 g/dL, as previously reported [28]. A serum albumin concentration of < 2.7 g/dL was considered as confirming the presence of PLE [29]. Exceeding serum was then frozen-stored at − 80 °C until analysis.

The clinical severity of the disease was evaluated using the previously published CCECAI scores [30]. The CCECAI score included the following clinical signs and laboratory evaluations: attitude/activity, appetite, vomiting, stool consistency, stool frequency, weight loss, serum albumin concentration, peripheral edema or ascites, and severity of pruritus [30]. The CCECAI score was assessed for each dog at the time of endoscopy (T0).

CCECAI was also calculated at 1-month (T1) and 6-month (T6) after the initiation of the immunosuppressant treatment. At T1, the IRE dogs were classified as complete responders (decreased CCECAI > 75% compared to T0), partial responders (decreased CCECAI of 25–75%) and non-responders (decreased CCECAI < 25%). At T6, the dogs considered responders at T1 were classified as relapsed (T6 CCECAI > 3 and an increase of at least 2 score points compared to T1) or non-relapsed (CCECAI < 3 or an increase of < 2 score points compared to T1). Finally, dogs that died from an IRE-related cause were included in the non-responder or relapsed groups, if deceased before T1 and between T1 and T6, respectively.

Only IRE dogs fed with the same hydrolyzed diet (HA Hydrolyzed®, Purina, Nestle Italia S.p.a, Mantova, Italy) for at least 2 weeks before diagnosis and during the study period were included. At the time of diagnosis, the Body Condition Score (BCS, score range 1–9) was evaluated in all dogs. They all also underwent immunosuppressant therapy (one or more between: prednisolone, budesonide, chlorambucil, cyclosporine, azathioprine). However, no therapeutic protocols have been reviewed.

### *Control dogs*

Twenty-six control dogs (CD) were included with their owners’ consent. Client consent was obtained for client-owned dogs of various breeds and ages. The CD were healthy dogs recruited during blood donation. All CD dogs were found to be clinically healthy from the physical examination, with no historical complaints and normal blood parameters. Exceeding serum from healthy donor dogs, with at least 8 h of fasting, is routinely stored at − 80 °C for scientific purposes and was used for our study for AAs determination. To be included in this study, all the CD has to be fed a maintenance diet for adult dogs (Purina® or Royal Canin®) and BCS has to be evaluated in all dogs.

### *Serum amino acid evaluation*

In all patients (CD and IRE group), the following 19 serum AA concentrations (nmol/mL) were evaluated on frozen-stored serum samples: L-Serine (SER), L-Aspartic acid (ASP), L-Glutamic acid (GLU), Glycine, L-Histidine (HIS), L-Arginine (ARG), L-Threonine (THR), L-Alanine (ALA), Proline (PRO), L-Amino-n-butyric acid (AABA), L-Cysteine (CYS), L-Tyrosine (TYR), L-Valine (VAL), Glycine (GLY), L-Methionine (MET), L-Lysine (LYS), L-Isoleucine (ILE), L-Leucine (LEU), L-Phenylalanine (PHE), Tryptophan (TRP).

These were analyzed with an automated high-performance liquid chromatography (HPLC) amino acid analyzer (AAA AccQ-Tag Test®, Merck Millipore Corporation). The samples for AA analysis were stored at − 80 °C both before and after deproteinization and functionalization and defrost only shortly before use and acquisition, the serum must be rapidly deproteinated before the functionalization to avoid interference. The reagent kit consists of Waters AccQ-tag borate buffer, Waters AccQ Fluor reagent powder (6-aminoquinolyl-N-hydroxy-succinimidyl carbamate), Waters AccQ Fluor reagent diluent, Waters Amino Acid Hydrolysate Standard (each ampoule contains a 2.5 mM mixture of the 19 hydrolysate amino acids).

For the preparation of the reagent, 1.0 ml of AccQ Fluor reagent diluent was transferred into a vial containing Waters AccQ Fluor reagent powder. This closed vial was mixed using a vortex for 10 s and heated on a heating block (55 °C) until it dissolved. The dissolved reagent can be refrigerated for up to 2 weeks. To ensure the preparation was of the internal calibration standard AAbA, 40 μl amino acid hydrolysate, 40 μl internal standard stock solution (6.45 mg α-aminobutyric acid to 25 ml 0.1 M HCl), 920 μl water HPLC grade were mixed with the vortex for 10 s. The derivatization procedure involved preheating of the heating block to 55 °C and mixing 10 μl of the calibration standard 70 μl AccQ Fluor borate buffer in a tube and 20 μl of the reconstituted AccQ Fluor reagent and mixing immediately for several seconds with the vortex. It was then heated in the heating block at 55 °C for 10 min (10 μl of the derivatized standard contained 50 pmol of each amino acid except cystine at 25 pmol).

The free amino acids in the serum were analyzed through three steps: 1) the deproteination of the serum; 2) the functionalization of free amino acids; and 3) the quantification of free amino acids. Deproteination was conducted using 100 μl of serum added to 100 μl of 10% aqueous TFA (trifluoroacetic acid), centrifuged for 10 min at 4400 g at 4 °C. The supernatant was then separated from the pellet, and the free amino acids were determined on the supernatant. For the functionalization, 5 μl of the deproteinated sample, 5 μl of internal standard 5 μl of 0.5 M NaOH, 60 μl of the borate buffer and 20 μl of Waters AccQ Fluor reagent were mixed for a few seconds and kept at room temperature for 1 min. The mixture was then heated to 55 °C for 10 min to hydrolyze the excess reagent.

The HPLC analytical procedure for quantifying free AAs was conducted using a DAD detector at 254 nm. The column matrix was RP18 (Nova-Pak C18, 4 μm, 150 × 3.9 mm) and the analysis was conducted with a constant flow of 1 ml/min. The column was thermostated at 37 °C, and the injection volume was 10 μl. The mobile phase A consisted of an aqueous buffer (supplied with the AccQ-Tag kit and prepared from 200 ml Waters AccQ Tag Eluent A concentrate added to 2 l of HPLC grade water and mixed), the mobile phase B was HPLC grade acetonitrile and the mobile phase C was HPLC grade water (see Table 2).

### *Statistical analysis*

The continuous variables were analyzed with the D’Agostino-Pearson normality test and expressed as median and range if non-parametric, and as mean ± standard deviation if normally distributed. Age and sex were compared between IRE and CD dogs using Mann-Whitney U-test and Fisher’s exact test, respectively.

Each AA was compared in CD and IRE dogs using the unpaired Mann-Whitney U-test or an unpaired t-test, depending on the D’Agostino-Pearson normality test. IRE dogs were divided in groups based on age categories (< 2 years, 2–7 years and > 7 years), sex (male/female), BCS score (2–6), serum total protein (hypoproteinemia/non-), serum albumin (PLE/non-), T1 CCECAI (responders/non-) and T6 CCECAI (relapsed/non-). The serum AAs of the two groups were compared using the Mann-Whitney U-test or the unpaired t-test and between three or more groups with the Kruskal-Wallis or one-way ANOVA tests followed by Bonferroni post-hoc test. Finally, each amino acid was correlated with total protein, albumin serum levels and CCECAI score using Pearson’s or Spearman’s correlation tests, based on the normality distribution (GraphPad Prism 6 for Mac, GraphPad Inc., La Jolla, CA). A *p*-value of < 0.05 was considered significant.

## Data Availability

The datasets used and/or analysed during the current study are available from the corresponding author on reasonable request.

## Abbreviations

AA: amino acid
AABA: L-Amino-n-butyric acid
ARG: L-Arginine
ASP: L-Aspartic acid
BCS: body condition score
CCECAI: chronic enteropathy clinical activity index
CD: control dogs
CYS: L-Cysteine
GLY: Glycine
GLU: L-Glutamic acid
HIS: L-Histidine
HPLC: high-performance liquid chromatography
IBD: inflammatory bowel disease
ILE: L-Isoleucine
IRE: immunosuppressant responsive enteropathy
LEU: L-Leucine
LYS: L-Lysine
MET: L-Methionine
PHE: L-Phenylalanine
PLE: protein-losing enteropathy
PRO: Proline
SER: L-Serine
THR: L-Threonine
TRP: tryptophan
TYR: L-Tyrosine
VAL: L-Valine
